# Supplementary material for: Primary glia cells from bank vole propagate multiple rodent-adapted scrapie prions
Source: Sci Rep. 2022 Feb 9;12:2190. doi: 10.1038/s41598-022-06198-4 (PMC8828835; doi:10.1038/s41598-022-06198-4)
Supplement: Supplementary file 1 — Supplementary Information. [file 41598_2022_6198_MOESM1_ESM.pdf]

Primary glia cells from bank vole propagate multiple rodent-adapted scrapie prions

Karla A. Schwenke, Joo-Hee Wälzlein, Agnieszka Bauer, Achim Thomzig, Michael Beekes

Supplementary Information

1) Relative Quantification of proteinase-resistant PrP in Initial Inocula

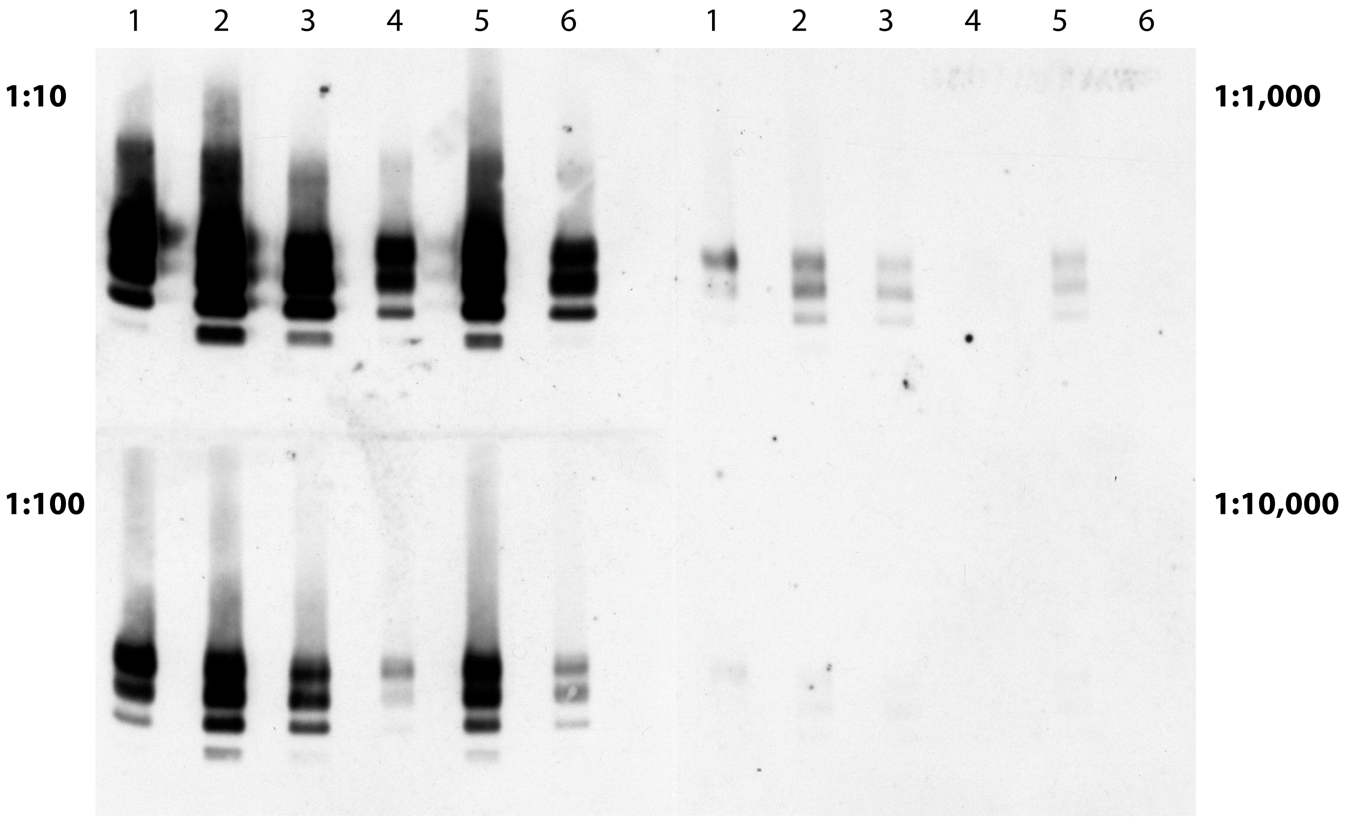

1 – 263K
2 – BL6-22L
3 – BL6-RML
4 – BV-RML
5 – BL6-ME7
6 – BV-ME7

Inocula as 10% (w/v) brain homogenates, proteinase K-treated (75 µg/ml, 37 °C, 1h), diluted in Sample Loading Buffer as indicated. Lanes between samples were left blank.

mAb: ICSM-18 (1:4,000)

2) Proteinase K digestion of cell lysates with lower concentrations

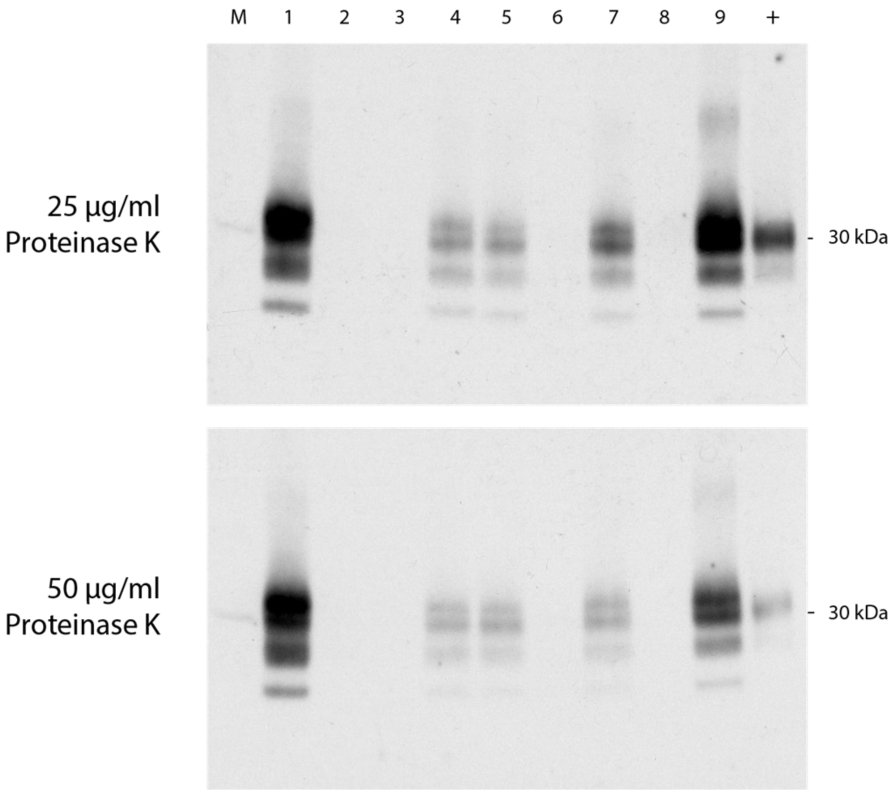

Cell lysates 60 dpe

1 - BL6-Glia + BL6-22L
2 - BL6-Glia + BL6-RML
3 - BL6-Glia + BL6-ME7
4 - BV-Glia + BL6-22L
5 - BV-Glia + BL6-RML
6 - BV-Glia + BL6-ME7
7 - BV-Glia + BV-RML
8 - BV-Glia + BV-ME7
9 - BV-Glia + Ha-263K

\+ 263K 10% brain homogenate (1:1,000)
M Low Molecular Weight Marker

PK digestion: 25 or 50 µg/ml PK, 37 °C, 1 h
mAb: ICSM-18 (1:4,000)

3) Untreated and Proteinase K-treated cell lysates

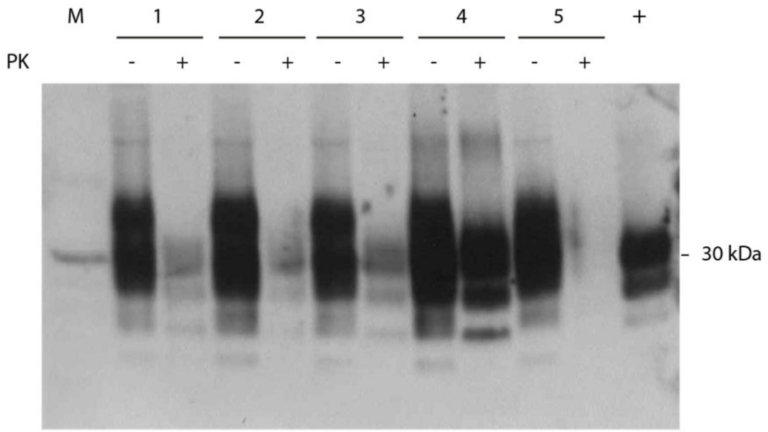

BV-Glia cell lysates at 60 dpe

1 - BL6-22L
2 - BL6-RML
3 - BV-RML
4 - Ha-263K
5 - NBH

\+ 263K 10% brain homogenate (1:1,000)
M Low Molecular Weight Marker

PK digestion: 75µg/ml PK, 37 °C, 1 h
mAb: ICSM-18 (1:4,000)

4) BV-Glia infected with 263K – Comparison of mAb ICSM-18 and 3F4

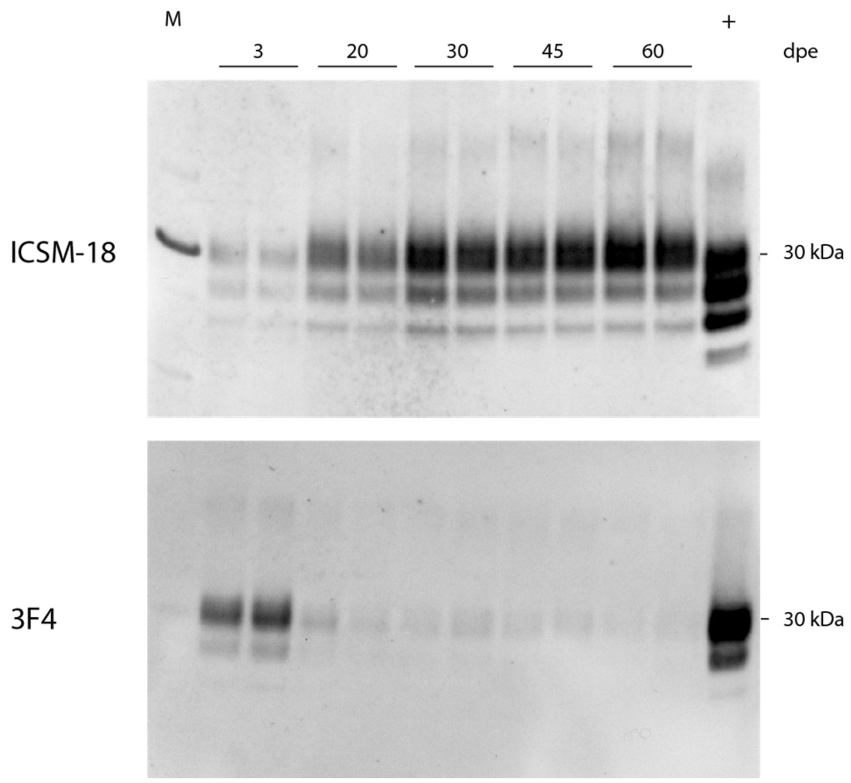

Samples blotted with

- mAb ICSM-18 (1:4,000) for murine PrP
- mAb 3F4 (1:2,000) for hamster PrP (of residual inoculum)

\+ - 139A (1:100) for ICSM-18
\+ - 263K (1:1,000) for 3F4

5) Comparison of relative PrP^{C} levels in glia cells and bank vole brain homogenate

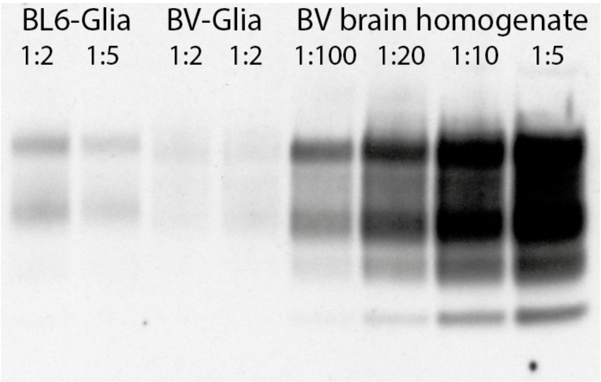

Dilutions of cell lysates of uninfected BL6-Glia and BV-Glia (each confluently grown 6-wells lysed in 100 µl Sarcosyl) in comparison to a diluted 10% bank vole-brain homogenate

- Dilutions in sample loading buffer as stated
- 10 µl per dilution loaded on gel
- Blot stained with mAb ICSM-18 (1:4,000)

→ BL6-Glia exhibit more PrP^{C} than the BV-Glia

6) BV-Glia infected with BV-RML – all replicate experiments

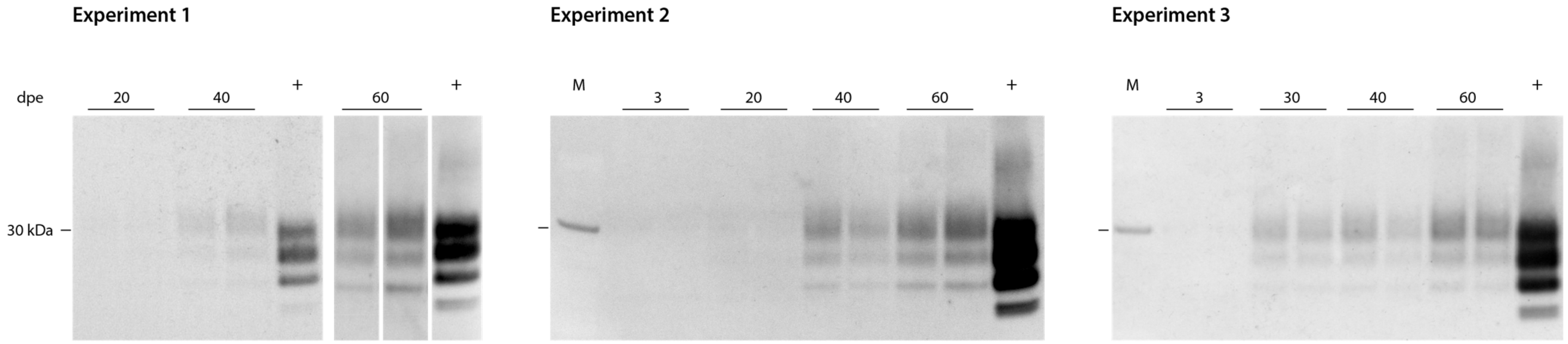

M Low Molecular Weight Marker
\+ 139A 10% brain homogenate (1:100)

PK digestion: 75 µg/ml PK, 37 °C, 1 h
mAb: ICSM-18 (1:4,000)

7) Complete Blots of Figure 2

Fig. 2 A

BL6-Glia infected with BL6-22L
3-60 dpe

M + [1]

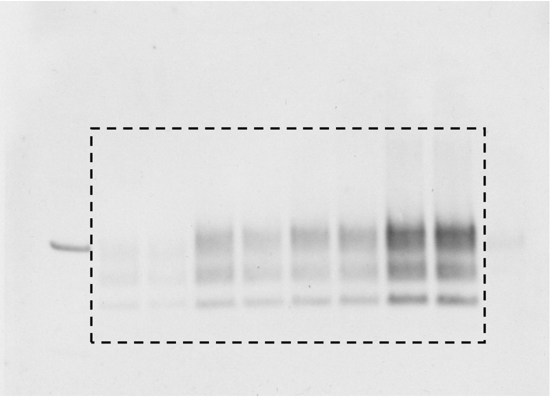

BL6-Glia infected with BL6-22L
60 dpe, deglycosylated (-/+)

M + [2]

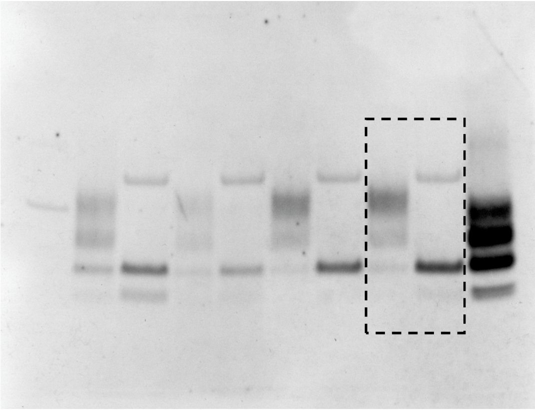

Fig. 2 B

BL6-Glia infected with BL6-RML
3-60 dpe

M + [1]

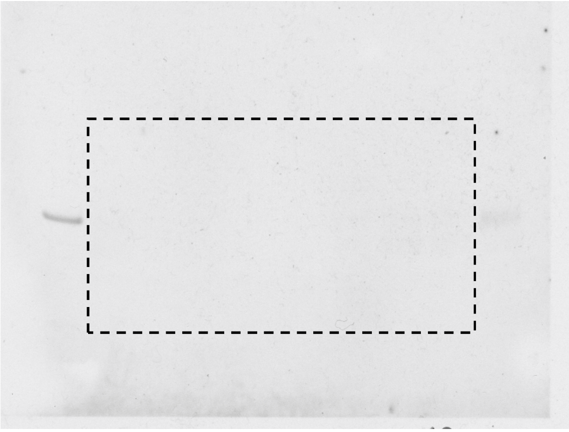

M – Low Molecular Weight Marker
+ – Loading control: 263K [1] / 139A [2] 10% brain homogenate (diluted 10^{-4} / 10^{-2})

7) Complete Blots of Figure 2

Fig. 2 C

BV-Glia infected with BL6-22L
3-60 dpe

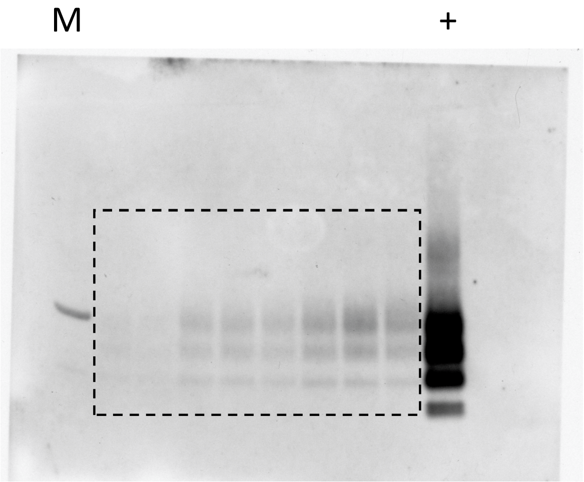

BV-Glia infected with BL6-22L
60 dpe, deglycosylated (-/+)

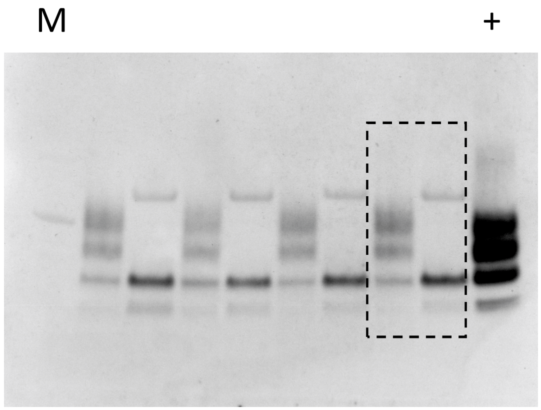

Fig. 2 D

BV-Glia infected with BL6-RML
3-60 dpe

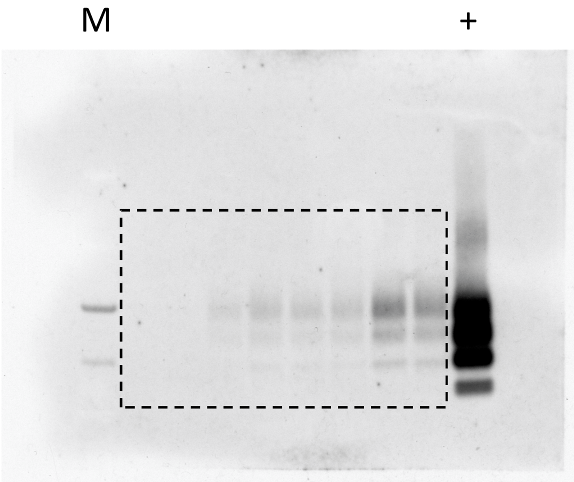

M – Low Molecular Weight Marker
+ – Loading control: 139A 10% brain homogenate (1:100)

7) Complete Blots of Figure 2

Fig. 2 E

BV-Glia infected with BV-RML
3-60 dpe

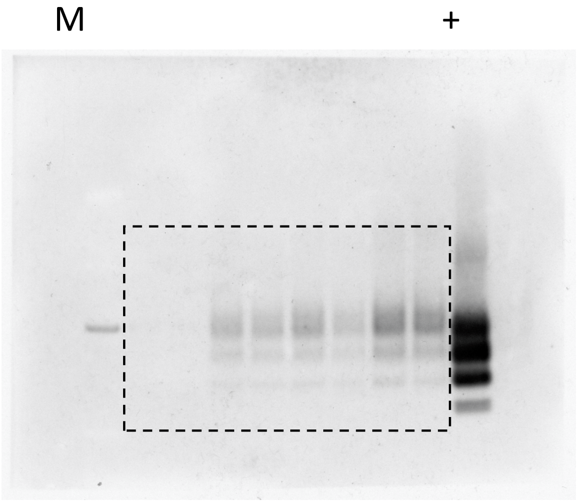

BV-Glia infected with BV-RML
60 dpe, deglycosylated (-/+)

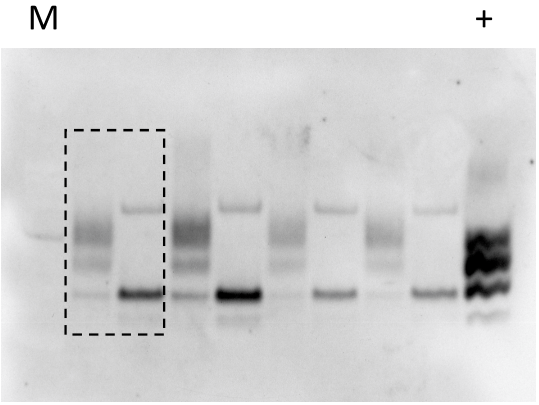

Fig. 2 F

BV-Glia infected with 263K
3-60 dpe

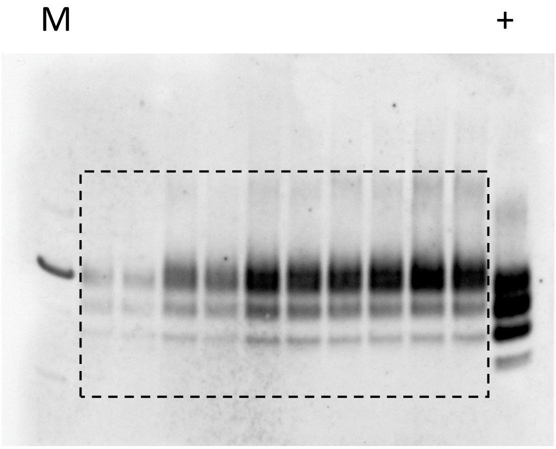

M – Low Molecular Weight Marker
\+ – Loading control: 139A 10% brain homogenate (1:100)
